# Evaluation of Photoplethysmography-Based Monitoring of Respiration Rate During High-Intensity Interval Training: Implications for Healthcare Monitoring

**DOI:** 10.3390/bios14120631

**Published:** 2024-12-20

**Authors:** Marjolein Muller, Kambiz Ebrahimkheil, Tara Vijgeboom, Casper van Eijck, Eelko Ronner

**Affiliations:** 1 Corsano Health B.V., Wilhelmina van Pruisenweg 35, 2595 AN The Hague, The Netherlands; 2 Department of Cardiology, Reinier de Graaf Hospital, Reinier de Graafweg 5, 2625 AD Delft, The Netherlands

**Keywords:** respiration rate, photoplethysmography, continuous monitoring, high-intensity interval training, remote monitoring

## Abstract

Monitoring respiration rate (RR) is crucial in various healthcare settings, particularly during demanding (physical) activities where respiratory dynamics are critical indicators of health status. This study aimed to evaluate the accuracy of photoplethysmography (PPG)-based monitoring of RR during high-intensity interval training (HIIT) and its potential applications in healthcare. Between January and March 2024, healthy volunteers participated in a cycling HIIT session with increasing resistance levels. The RR measurements obtained using the PPG-based CardioWatch 287-2 (Corsano Health) were compared with an ECG patch-derived (Vivalink) reference. Subgroup analyses were conducted based on skin type and sex. A total of 35 participants contributed 1794 paired RR measurements. The PPG algorithm for RR monitoring showed an average root mean square (Arms) error of 2.13 breaths per minute (brpm), a bias of −0.09 brpm, and limits of agreement (LoA) from −4.28 to 4.09 brpm. Results were consistent across the different demographic subgroups. The CardioWatch 287-2 therefore demonstrated reliable RR monitoring during HIIT, supporting its potential use in healthcare settings for continuous, non-invasive respiratory monitoring, particularly in physical rehabilitation and chronic respiratory condition management.

## 1. Introduction

In healthcare, accurately monitoring respiration rate (RR) is essential for evaluating a patient’s respiratory function, especially during activities that place high demands on the respiratory system [1]. RR is a critical vital sign that reflects the body’s ability to meet oxygen requirements, both at rest and during physical exertion. Accurate RR monitoring is therefore crucial across a range of healthcare applications, including pulmonary rehabilitation, chronic obstructive pulmonary disease (COPD) management, fitness assessments, and the monitoring of patients in both acute and chronic care settings [2,3].

The ability to obtain real-time, precise RR measurements becomes even more vital when individuals are engaged in physical activities, as these situations require the body to adjust its respiratory function rapidly [4]. Accurate, real-time RR data can provide healthcare providers insights into a patient’s physiological state, enabling timely and informed interventions [4,5]. For example, in pulmonary rehabilitation, continuously tracking RR allows for adjustments to exercise intensity, helping to keep patients within safe respiratory limits while optimizing therapeutic outcomes [2,4].

Despite its clinical importance, RR remains under-monitored due to the lack of accessible, non-invasive, and reliable methods, particularly during dynamic activities [1,2]. This is concerning because RR can be a sensitive indicator of physiological distress, particularly during physical exertion or in patients with respiratory conditions [2]. In fact, abnormal RR often precedes changes in other vital signs, making it an invaluable tool for early detection of deterioration in a patient’s condition [4].

To address this challenge, wearable technologies have increasingly turned to photoplethysmography (PPG), a method that measures light absorption by blood vessels to track changes in blood flow and oxygenation. PPG-based wearables, such as wristbands, offer a convenient and non-invasive way to monitor vital signs, including RR, in real time. However, despite the promise of this technology, many existing devices struggle with maintaining accuracy during motion-heavy activities, such as high-intensity interval training (HIIT), where physiological parameters fluctuate rapidly [6,7]. Current wearable devices often lack robust mechanisms to compensate for motion artifacts, leading to significant inaccuracies during activities involving dynamic movements. RR, in particular, is very difficult to monitor accurately due to the overlap between respiratory signals and noise, especially during motion [8]. This overlap makes it harder to differentiate true respiratory changes from artifacts caused by movement, leading to potential inaccuracies in the data and risk of inappropriate clinical decisions.

The demand for reliable RR monitoring is particularly relevant in contexts such as pulmonary rehabilitation, fitness assessment, and the management of chronic conditions like COPD. In these applications, continuous and accurate RR data can guide clinical decisions and optimize therapy. Yet, most PPG-based devices face significant challenges in mitigating motion artifacts and maintaining accuracy across diverse populations. Furthermore, few studies have systematically evaluated these devices in high-motion scenarios, highlighting a critical gap in the literature.

Therefore, this study evaluates the accuracy of RR measurements provided by the Corsano CardioWatch 287-2, a PPG-based wristband equipped with advanced signal processing capabilities, during HIIT. This research incorporates a diverse participant pool, including individuals across the Fitzpatrick skin tone spectrum and both sexes, to evaluate the generalizability of the device. Additionally, the study leverages the CardioWatch’s novel time-frequency algorithm and multi-wavelength LEDs, technologies designed to address key challenges in motion-heavy and diverse-use contexts. By assessing the reliability of RR monitoring during such challenging activity, the study seeks to generate valuable insights that can inform healthcare strategies for a wide range of patients—including those involved in high-intensity exercise and individuals with respiratory or cardiovascular conditions who may benefit from continuous, non-invasive monitoring of their respiratory function, contributing valuable insights into the potential of wearable technology for both fitness and clinical applications.

## 2. Materials and Methods

This study was a single-center, non-randomized interventional trial conducted at The Hague Tech Centre (The Hague, The Netherlands) from January 2024 to March 2024. The research focused on evaluating the accuracy of the Corsano CardioWatch 287-2 [9] in monitoring RR during HIIT among healthy volunteers. Each participant attended a single session, where the RR data collected by the Corsano CardioWatch was compared with data from a reference device, the Vivalink wearable ECG patch (Meudon, France) [10].

The RR evaluation formed part of a broader study registered under NL.85330.058.23 (ToetsingOnline.nl), which also assessed pulse rate, oxygen saturation, interbeat intervals, and non-invasive blood pressure. The study was conducted in accordance with the Declaration of Helsinki, and the protocol was approved by the Ethics Committee of Leiden-Den Haag-Delft (P23.093) on 22 January 2024.

Participants included healthy adults aged 18 and older, with a distribution ensuring at least 30% male and 30% female, and 30% of participants were required to have a Fitzpatrick scale score of V or VI to ensure diversity in skin tones [11]. Exclusion criteria ruled out individuals with a baseline systolic blood pressure ≥160 mmHg, diastolic blood pressure ≥100 mmHg, high total cardiovascular risk, or any clinical condition posing risks during HIIT. Additionally, participants unable to wear the Corsano CardioWatch 287-2 due to factors such as allergic reactions, wounds, or amputations or those unwilling to provide informed consent were excluded. Recruitment was carried out via media platforms associated with The Hague Tech Centre, and all participants provided written informed consent before participating.

The accuracy of RR measurements from the Corsano CardioWatch 287-2 was compared to those from the Vivalink wearable ECG patch monitor across all participants during HIIT sessions. HIIT was chosen because its intense, fluctuating nature presents a rigorous test of the device’s performance under real-world conditions. A physician was present during each session to supervise and ensure participant safety. Each participant underwent a single, 20-min measurement session, during which the Corsano CardioWatch was worn on the right wrist, and the Vivalink ECG patch was placed on the left side of the chest as per standard instructions. Participants engaged in a HIIT protocol on a LifeSpan R3i stationary bike (Rotterdam, the Netherlands) [12], involving a warm-up, incremental increases in bike resistance to achieve specific heart rate ranges, and a cooldown period. Specifically, the following study protocol was applied: 2-min warm-up (own pace); increase pace/bike resistance to achieve 5 heartbeat ranges (120–130 beats per minute (bpm); 130–140 bpm; 140–160 bpm; >160 bpm) and hold each range for 30 s; and 2-min cooldown (own pace).

Baseline characteristics such as age, sex, weight, height, and skin color were recorded before the measurement session.

The investigational device, CardioWatch 287-2 [9], employed PPG for non-invasive monitoring of blood volume changes in the microvascular bed on the dorsal side of the wrist. The PPG sensor operated with two photodiodes that capture light reflected from the skin, using a proprietary time-frequency algorithm to extract respiratory-induced variations in signal intensity, amplitude, and frequency. This enabled high-precision RR assessment by providing detailed insights into respiratory patterns.

Data was wirelessly transmitted via Bluetooth to the Corsano app on a mobile device and securely uploaded to a health cloud and research portal. The device used six light-emitting diodes (LEDs)—two red, two green, and two infrared—to enhance PPG signal quality by capturing signals at different skin depths. Green light effectively reduced motion artifacts, while infrared light penetrated deeper for comprehensive measurements. An ambient light detection system mitigated interference from external light sources, ensuring signal integrity. Baseline wander caused by ambient light changes was corrected, normalizing the pulsatile blood flow signal for algorithm input.

The device dynamically adjusted LED power: increasing power if PPG values fell below a threshold and decreasing it when values exceeded the threshold. To ensure reliable data in dynamic conditions, an accelerometer (ACC) compensated for motion artifacts caused by user movement or external vibrations. The RR algorithm integrated both time-domain and frequency-domain analyses. The time-domain analysis was used in the absence of motion (as detected by the ACC), relying on amplitude and frequency modulation.

The frequency-domain analysis was activated during motion, leveraging the ACC signal to separate motion-related peaks from the respiratory peak in the PPG frequency domain. The ACC also assessed movement levels, assigning a quality factor to the PPG signal and RR prediction. Data was categorized into four movement levels: rest, mild movement, moderate movement, and heavy movement, with the latter excluded from analysis.

Both the PPG and ACC signals were measured at a frequency of 32 Hz. The proprietary algorithm processed these signals and generated an updated RR reading every 30 s. To mitigate transient fluctuations and noise, the RR data was averaged over this period, providing smoother pulse rate measurements and improving the stability of the results. Moreover, to avoid low signal quality, a signal qualifier was applied to remove low-quality parts of PPG recordings, called “de-noising of the signal.” The raw signals were de-noised by passing them through a low-pass filter, eliminating high-frequency noise from the signal. Finally, the algorithm filtered out the frequency corresponding to the resting heart rate to ensure that low resting heart rate frequencies were not included when determining RR.

RR measurements were paired between the two devices every 30 s throughout the entire study duration. The continuous data from the CardioWatch 287-2 was synchronized with the ECG patch by aligning the timestamps of the pulse oximeter with those of the Corsano mobile app, which was connected to both the CardioWatch 287-2 and the ECG patch. No time drift was observed between the devices during this process.

The PPG data from the CardioWatch was then pre-processed using a bandpass filter with a frequency range of 0.5 to 5 Hz to isolate the relevant respiratory signals. Following this, each pulse was evaluated for signal quality, and any pulses with a poor signal-to-noise ratio were removed to ensure accuracy.

To compare the RR values obtained from the Corsano CardioWatch 287-2 and the Vivalink ECG patch, the average root mean square error (Arms) was calculated. The primary endpoint was the Arms of RR monitoring, aiming for an Arms ≤4 breaths per minute (brpm).

The Arms was calculated using the following formula:

(1) $$Arms=\sqrt{\frac{\sum_{i=1}^{n}{\left({CW2}_{i}-R_{i}\right)}^{2}}{n}}$$

where *R_i_* represents the reference value for sample *i*, *CW2*_*i*_ denotes the value computed by the CardioWatch 287-2 software for sample *i*, and *n* is the total number of samples.

The Arms metric captures the bias (defined as the difference between the test results and an accepted reference value) and the precision (the closeness of agreement between independent test results obtained under stipulated conditions). Bias helps us to evaluate whether there is a systemic over- or underestimation in the predictors, whereas precision refers to the variability of errors. High precision indicates that predictions are consistent.

Bias and 95% limits of agreement (LoA) were also calculated separately based on the method by Bland et al., considering both within and between-subject variance [13]. Bland–Altman plots were constructed to visually assess the agreement between the two measurement methods by showing the differences and LoA, helping to identify systematic bias and outliers. These plots are simple to interpret, provide insights into precision, and do not require assumptions about data distribution. The LoA are typically set at ±1.96 standard deviations from the mean difference. These limits help quantify the range within which most differences between the two methods will lie. In other words, they define the interval where the differences between the two methods can be expected to fall for 95% of the subjects or samples.

The 95% confidence interval (CI) for pooled analysis bias and LoA was also determined, adhering to guidelines for multiple measurements per participant [14,15]. The CI provides a measure of the precision of the estimated bias and LoA, indicating the range within which the true values are likely to fall. This helps assess the reliability and statistical significance of the results.

To further evaluate the effect of motion on the performance of the RR algorithm, Arms and bias were additionally reported separately for data points recorded at rest, during mild motion, and during moderate motion. Heavy motion was not included, as this led to exclusion of the data points.

As sub-analyses, all primary endpoint calculations were performed separately for each sex category (male/female) and each skin color category based on the Fitzpatrick scale (I–IV and V–VI). A post-hoc power analysis was conducted using the G*Power tool (version 3.1.9.7) to determine the statistical power of these subgroup analyses. The analysis, based on the amount of included measurement pairs, evaluated the two-tailed difference in mean Arms between the two independent groups, with a significance level of 0.05 and a desired power of at least 80%.

## 3. Results

A total of 35 healthy volunteers completed the HIIT protocol as part of the study. The demographic characteristics of these participants are summarized in Table 1. Most participants were young and had relatively high fitness levels, although two older adults aged 58 and 60, with reduced fitness levels, were also included. There were no adverse or unexpected events during the study, and all participants completed the protocol without premature discontinuation. Additionally, no participants were excluded from the analysis due to poor data quality or technical issues, ensuring the integrity of the dataset.

### 3.1. Respiration Rate

#### 3.1.1. Primary Analysis

The accuracy of the RR algorithm implemented in the Corsano CardioWatch 287-2 was compared to the Vivalink ECG patch for 1794 data points (Table 2). The Arms value pooled across all data points was 2.13 brpm. The overall bias was nearly zero, with a mean percentage difference of −0.09 brpm. The 95% CI for this bias ranged from −0.19 brpm to 0.00 brpm, indicating a negligible deviation between the two devices.

**Figure 1:**
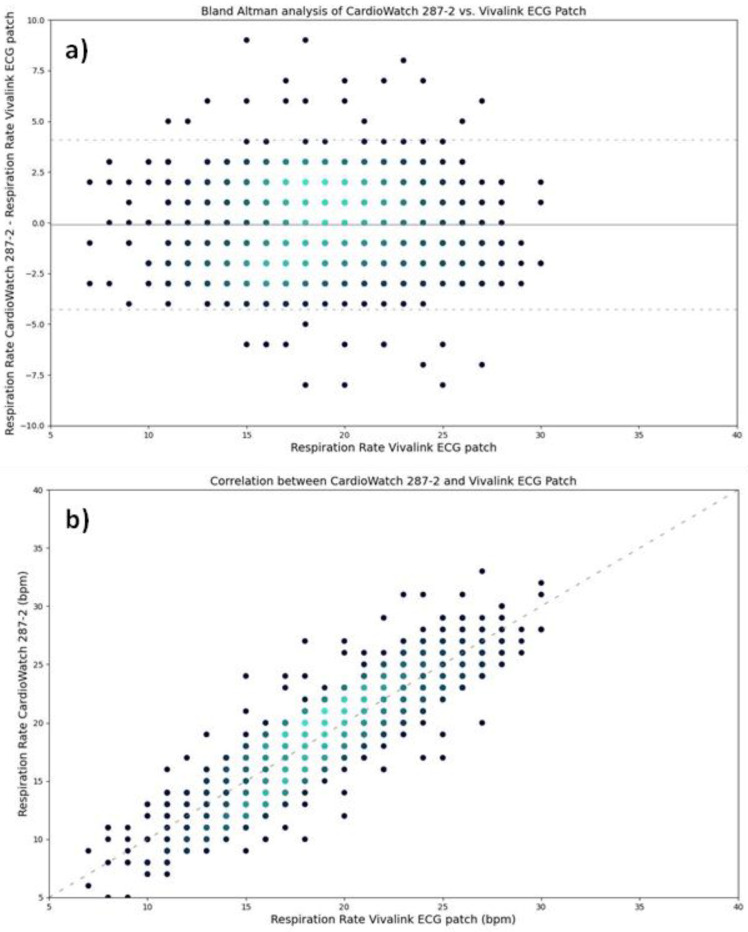
Comparison of CardioWatch 287-2 RR and Vivalink ECG Patch reference RR: (**a**) Bland–Altman Analysis: This plot illustrates the differences between the investigational device and the reference standard across the range of reference RRs. The mean bias was negligible (−0.09 brpm, 95% CI: −0.19 to 0.00), indicating no significant systemic bias. The LoA ranged from −4.28 brpm (95% CI: −4.37 to −4.17) to 4.09 brpm (95% CI: 3.99 to 4.19). Several instances of differences fall beyond these limits, though the distribution of differences does not exhibit a systematic trend relative to reference values. Density plots reveal clustering of data points, with higher density (lighter blue) around the mean difference. (**b**) Correlation Analysis: This plot shows a strong linear correlation between investigational and reference RRs, suggesting good overall agreement. Deviations from the ideal line of equality (dashed y = x line) are observed; however, this deviation is similar across all RRs. The density plot further highlights a high frequency of moderate RRs (15–25 bpm). Density plot where high density is shown in light blue and lower density in dark blue.

Bland–Altman analysis was performed to evaluate the LoA (Figure 1). The lower LoA was found to be −4.28 brpm (95% CI: −4.37 brpm to −4.17 brpm), and the upper LoA was 4.09 brpm (95% CI: 3.99 brpm to 4.19 brpm).

**Figure 2:**
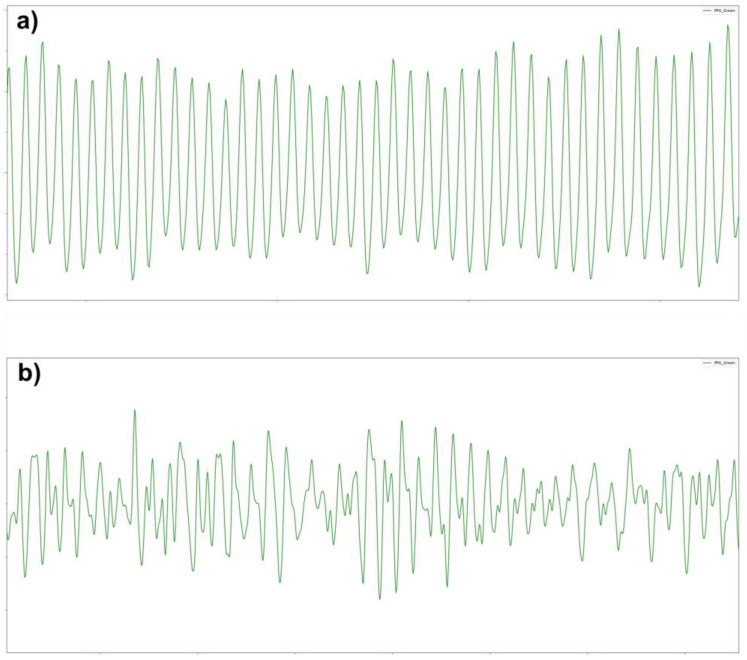
Comparison of CardioWatch 287-2 PPG signals during (**a**) resting state and (**b**) moderate motion. The images highlight the significant alterations in the PPG signal caused by motion. Despite these changes, the PPG signal during moderate motion retains the respiratory frequency, allowing for the extraction of RR. This is achieved by leveraging frequency-domain data from the accelerometer to correct for motion artifacts and isolate the non-motion-related frequency corresponding to respiration.

The Arms and bias of the RR algorithm slow slight changes due to motion (Table 3). When comparing the performance of the RR algorithm across data points recorded during rest, mild motion, or moderate motion, the Arms increases from 1.5 brpm at rest to 2.4 brpm at moderate motion. The RR algorithm demonstrates a positive bias (0.25 brpm) at rest, whereas it becomes negative at mild and moderate motions (−0.56 brpm and −0.12 brpm, respectively). These changes occur due to an increased noise level during motion (Figure 2).

#### 3.1.2. Sub-Analyses

The results of the sub-analyses comparing subgroups based on sex and skin color were consistent with those observed for the pooled dataset. The Arms values for the subgroups ranged from 2.02 to 2.20 brpm, which is similar to the Arms value of 2.13 brpm for the pooled data. Specifically, the Arms for the male patient subgroup was 2.20 brpm, while for the female subgroup, it was slightly lower at 2.02 brpm. The power for this subgroup analysis was adequate (≥80%) based on a post-hoc power analysis, which considered the sample sizes and a Cohen’s d of 0.98 for the average Arms and its standard deviation.

Bias and LoA were found to be slightly lower for the female subgroup compared to the male subgroup, as detailed in Table 4.

For patients with a skin color within Fitzpatrick scale I–IV, the Arms was 2.11 brpm, whereas for patients with a skin color within Fitzpatrick scale V–VI, the Arms was slightly higher at 2.20 brpm. The power for this subgroup analysis was adequate (≥80%) based on a post-hoc power analysis, which considered the sample sizes and a Cohen’s d of 0.46 for the average Arms and its standard deviation.

The corresponding bias and LoA were also slightly lower for the subgroup with Fitzpatrick scale I–IV compared to the subgroup with Fitzpatrick scale V–VI, as shown in Table 5.

## 4. Discussion

The findings of this study contribute to a growing body of research demonstrating the potential of wearable technology, specifically PPG-based devices, in accurately monitoring RR during HIIT. The Corsano CardioWatch 287-2 showed promising results when compared to the Vivalink ECG patch. In fact, it demonstrated an Arms of 2.13 brpm, which surpasses the accuracy threshold (Arms ≤ 4 brpm) typically deemed acceptable for wearable devices. Compared to the bias values reported for other devices in the literature [7,16], the minimal bias of −0.09 brpm highlights the strong agreement between the Corsano CardioWatch and the reference standard. Additionally, the low LoA observed in this study (−4.28 to 4.09 brpm) indicate a narrow range of error, further reinforcing the reliability of the device for practical applications. To our knowledge, these results are superior to those presented in earlier research [7,16].

The robustness of the Corsano CardioWatch 287-2 is further evidenced by its ability to handle motion artifacts effectively, a critical requirement during activities like HIIT, which involve rapid and intense movements. The inclusion of an accelerometer ensures that motion-induced noise is effectively filtered out using an adaptive filter, dynamically isolating true physiological signals from unwanted noise. This feature is complemented by the ambient light detection system, which corrects for baseline wander caused by external light sources, ensuring consistent signal quality. Together, these advancements make the CardioWatch 287-2 particularly well-suited for use in dynamic and physically demanding environments, rivaling the Vivalink ECG patch in performance.

These results align with and expand upon findings in the broader literature. Previous research has highlighted the challenges of RR monitoring in dynamic environments, particularly during high-intensity activities where motion artifacts and physiological variations complicate PPG signal interpretation [6,7]. The Corsano CardioWatch 287-2 effectively addresses these challenges with its advanced signal processing techniques, which are in line with improvements noted by Allen et al. (2021) and Charlton et al. (2022). They noted that improvements in signal processing have significantly enhanced the accuracy of wearable devices under dynamic conditions [17,18]. Similarly, Kazemi et al. (2024) discussed the integration of machine learning algorithms in wearable health technology, stating that it has further improved the ability to filter out noise and motion artifacts, thus enhancing the accuracy of RR measurements during exercise [19]. This aligns with the findings of this study, where the Corsano CardioWatch 287-2, equipped with a time-frequency algorithm, demonstrated high accuracy even during the rigorous demands of HIIT.

The potential of wearable devices for monitoring RR in clinical populations has also been explored in recent research. For example, studies by Joseph et al. (2022) and Lu et al. (2023) have investigated the use of wearable PPG devices in patients with chronic respiratory conditions such as COPD [20,21]. These studies found that while wearable technology shows promise, the accuracy of RR monitoring can vary depending on the severity of the condition and presence of comorbidities. This underscores the importance of validating devices like the Corsano CardioWatch 287-2 in broader and more diverse patient populations.

## 5. Limitations

Despite the promising results, several limitations of this study should be acknowledged. Firstly, the study was conducted in a controlled environment with a relatively small sample size of 35 participants. While the diversity in skin tone (as categorized by the Fitzpatrick scale) and the inclusion of both sexes add robustness to the findings, the sample may not fully represent the broader population, especially those with underlying respiratory or cardiovascular conditions. The small sample size limits the generalizability of the findings, and the inclusion of individuals with different levels of fitness or pre-existing conditions would provide a more comprehensive understanding of the device’s performance. Future studies should expand the sample size to increase statistical power and explore device performance across a more diverse set of demographic characteristics, including age groups and fitness levels.

Secondly, the study focused solely on healthy volunteers. The performance of the Corsano CardioWatch 287-2 in populations with compromised respiratory function, such as patients with COPD or heart failure, remains to be validated. This is particularly important as such populations are likely to benefit most from accurate and continuous RR monitoring. The first steps to evaluate the performance in clinical conditions have been made by Monnink et al. (2024) [22]; however, future studies should aim to further assess the device’s accuracy and reliability in clinical populations where accurate RR monitoring is critical for patient management. Additionally, exploring the device’s integration into rehabilitation programs or as part of continuous home monitoring systems in cardiac and pulmonary patients during exercise could strengthen its clinical relevance.

Thirdly, the study was limited to a single activity (cycling HIIT) in a controlled environment. While cycling involves relatively steady hand and arm movements, other activities such as running or team sports involve more variable motion, which could introduce additional artifacts and noise. Future research should evaluate the device across a range of physical activities and for a prolonged time period (hours up to days) to understand its robustness in real-world conditions. Similarly, testing the device in outdoor environments where ambient light and temperature fluctuations could impact performance would further validate its applicability.

Finally, the study used the Vivalink ECG patch as the reference standard, which, while reliable, is not the gold standard for RR measurement. Comparison with capnography, considered the gold standard for RR monitoring, would provide further validation of the CardioWatch 287-2’s accuracy, allowing a more definitive assessment of the device’s performance in both fitness and clinical settings.

## 6. Conclusions

This study demonstrates that the Corsano CardioWatch 287-2 wristband offers a reliable and accurate method for monitoring RR during high-intensity physical activity. The device’s performance, characterized by a low Arms value and minimal bias when compared to a reference device, suggests that it is well-suited for real-time monitoring in both fitness and potential clinical settings. Its advanced time-frequency algorithm and motion artifact compensation make it a reliable solution for dynamic environments, addressing key challenges in wearable technology. By including diverse participants in terms of skin tone and sex, the study also underscores the device’s broad applicability, filling critical gaps in existing PPG-based monitoring solutions. Future research should extend these findings to other physical activities and clinical populations, as well as compare the device with capnography to further validate its utility.

## Figures and Tables

**Table 1:** Demographics of enrolled and analyzed participants (*N* = 35).

Demographic	Mean (±SD)
Age, years	30 (±10)
Male, N (%)	22 (63%)
Weight, kg	73 (±11)
Height, cm	176 (±9)
Skin color (Fitzpatrick), N (%)	
Class I–II	11 (31%)
Class III–IV	13 (37%)
Class V–VI	11 (31%)

Abbreviations: SD: standard deviation; N: number of patients.

**Table 2:** Accuracy of RR measurements by the Corsano CardioWatch 287-2 compared to Vivalink ECG patch reference measurements, pooled across all subjects.

Outcome Measures	Pooled Results (*n* = 1794)
Arms [brpm]	2.13
Bias (+95% CI) [brpm]	−0.09 (−0.19, 0.00)
Lower 95% LoA (+95% CI) [brpm]	−4.28 (−4.37, −4.17)
Upper 95% LoA (+95% CI) [brpm]	4.09 (3.99, 4.19)

Abbreviations: brpm: breaths per minute; n: number of samples; Arms: Average root mean square; CI: confidence interval; LoA: limits of agreement.

**Table 3:** Accuracy of RR measurements by the Corsano CardioWatch 287-2 compared to Vivalink ECG patch reference measurements for recording during rest, mild motion, and moderate motion reported separately.

Activity Level	Arms [brpm]	Bias (+95% CI) [brpm]
Rest (*n* = 537)	1.5	0.25 (0.12, 0.38)
Mild motion (*n* = 337)	2.1	−0.56 (−0.35, −0.79)
Moderate motion (*n* = 920)	2.4	−0.12 (−0.27, 0.04)

Abbreviations: brpm: breaths per minute; *n*: number of samples; Arms: Average root mean square; CI: confidence interval.

**Table 4:** Accuracy of RR measurements by the Corsano CardioWatch 287-2 compared to Vivalink ECG patch reference measurements, for subgroups based on sex.

Demographics	Male (N = 22) Mean [95% CI]	Female (N = 13) Mean [95% CI]
Age, years	30.5 [26.3–34.8]	25.8 [20.2–31.3]
Weight, kg	79.2 [76.4–81.9]	62.9 [59.0–66.8]
Height, cm	181.6 [179.2–184.1]	170.0 [165.5–174.5]
Skin color (Fitzpatrick), N (%)		
Class I–II	4 (18.2%)	7 (53.8%)
Class III–IV	8 (36.4%)	5 (38.5%)
Class V–VI	10 (45.5%)	1 (7.7%)
**Outcome Measures**	**Male (*n* = 1102)**	**Female (*n* = 692)**
Arms (SD) [brpm]	2.20 (0.15)	2.02 (0.21)
Bias (+95% CI) [brpm]	−0.18 (−0.31, −0.05)	0.05 (−0.10, 0.20)
Lower 95% LoA (+95% CI) [brpm]	−4.48 (−4.61, −4.35)	−3.93 (−4.08, −3.77)
Upper 95% LoA (+95% CI) [brpm]	4.12 (3.99, 4.25)	4.02 (3.87, 4.18)

Abbreviations: brpm: breaths per minute; N: number of patients; CI: confidence interval; *n*: number of samples; Arms: Average root mean square; LoA: limits of agreement.

**Table 5:** Accuracy of RR measurements by the Corsano CardioWatch 287-2 compared to Vivalink ECG patch reference measurements, for subgroups based on skin color.

Demographics	Fitzpatrick I–IV (N = 24) Mean [95% CI]	Fitzpatrick V–VI (N = 11) Mean [95% CI]
Age, years	29.9 [25.3–34.4]	28.6 [24.7–32.6]
Male, N (%)	12 (50.0%)	10 (90.9%)
Weight, kg	70.2 [66.1–74.3]	79.6 [75.3–84.0]
Height, cm	175.9 [172.3–179.5]	181.5 [177.4–185.6]
**Outcome Measures**	**Fitzpatrick I–IV (*n* = 1258)**	**Fitzpatrick V–VI (*n* = 536)**
Arms (SD) [brpm]	2.11 (0.19)	2.20 (0.20)
Bias (+95% CI) [brpm]	−0.05 (−0.16, 0.06)	−0.20 (−0.38, −0.01)
Lower 95% LoA (+95% CI) [brpm]	−4.18 (−4.29, −4.06)	−4.49 (−4.68, −4.31)
Upper 95% LoA (+95% CI) [brpm]	4.08 (3.97, 4.20)	4.10 (3.92, 4.29)

Abbreviations: brpm: breaths per minute; N: number of patients; *n*: number of samples; Arms: Average root mean square; CI: confidence interval; LoA: limits of agreement.

## Data Availability

All data generated and analyzed during this study will be made available by the corresponding author upon reasonable request.
